# From platform carnival to official discourse: How “truth” is produced in Chinese online news

**DOI:** 10.1371/journal.pone.0348284

**Published:** 2026-06-10

**Authors:** Jiayu Li, Shiming Hu

**Affiliations:** 1 College of Music, Guangdong Polytechnic Normal University, Guangzhou, Guangdong, China; 2 School of Journalism and Communication, Zhengzhou University, Zhengzhou, Henan, China; Universiti Malaya, MALAYSIA

## Abstract

How is “truth” sustained amid algorithmic and political constraints? This study explores the process by which “truth” is negotiated and stabilized in China’s online journalism. Drawing on Actor-Network Theory (ANT) and controversy mapping, we translate three cases to illustrate how online controversies evolve from public affect to institutional closure. The findings reveal that in Chinese digital journalism, diverse human and nonhuman actors jointly participate in the construction of truth. Digital platforms such as Weibo function as obligatory passage points that mediate between public participation and state regulation. While user content amplifies emotion and scrutiny, algorithmic visibility and bureaucratic inscription serve to stabilize discourse. These interactions demonstrate that truth is not discovered but co-produced across technological, institutional, and affective domains. Consequently, Chinese online journalism can be conceptualized as a techno-cultural assemblage in which human agency, platform logic, and governmental governance are continuously negotiated in the production of news and facts.

## Introduction

In the digital communication environment, the relationship between news and truth has shifted from representing reality to negotiating reality. Digital platforms have reshaped the production, distribution, and reception of news. Early scholarship emphasized how online environments enabled new participatory forms of newswork, such as “gatewatching,” where audiences collaboratively monitor, filter, and circulate information [1]. At the same time, the conceptualization of sources in online news has been reconceptualized, as journalists negotiate authority with an expanded range of actors, including citizens, platforms, and algorithms [2,3]. Taken together, research related to online news underscores how the digital turn in journalism is simultaneously disrupting established routines and generating new forms of collaborative, networked news production.

The question of how truth emerges therefore remains a central issue surrounding journalistic practice, media construction, and the formation of social consensus. Journalism research generally assumes that truth gradually approaches reality through professional practices such as fact-checking, source verification, and sustained reporting [4]. However, as communication shifts from mass media systems to platform-based environments, research has increasingly adopted multidimensional perspectives, including social construction, discursive struggles, technological mediation, and power structures [5, 6]. More recently, under conditions characterized by post-truth politics, information epidemics, and deepfake technologies, scholars have examined the fragility of truth, crises of consensus, and the growing importance of platform responsibility and fact-checking mechanisms [7–10]. These developments have shifted attention from media-centered verification toward distributed, networked, and participatory processes of truth negotiation.

Within this broader transformation, China provides a particularly revealing context. Online news operates within a unique media landscape balancing state oversight with rapidly expanding public participation, making it a critical lens for observing how truth is negotiated in environments where institutional authority and platform-based participation coexist [11]. On the one hand, the state’s emphasis on guiding public opinion positions official discourse as a benchmark of truth, while censorship and propaganda practices aim to stabilize consensus [12]. On the other hand, platforms such as Weibo, WeChat, and Douyin function as arenas of participatory expression where news events are reframed, contested, and reinterpreted through user interaction [13]. The result is a hybrid communicative environment. Truth is neither solely imposed by institutions nor fully determined by online publics. Instead, it is continuously negotiated across platform infrastructures, journalistic mediation, and administrative interventions.

Despite the richness of existing scholarship, much of the literature tends to examine state-centered mechanisms of discourse control or online public participation separately. Less attention has been paid to the dynamic processes through which controversies migrate from decentralized platform discussions into authoritative institutional narratives. In other words, the mechanisms through which contested information becomes stabilized as publicly recognized truth remain insufficiently explored. As Waisbord observes, “truth is what happens to news [14].” However, the mechanisms through which truth becomes stabilized through news circulation remain insufficiently explored.

Actor-Network Theory (ANT) provides a framework capable of addressing this gap by conceptualizing news production as the outcome of interactions among heterogeneous human and non-human actors. A growing body of scholarship has demonstrated ANT’s utility in analyzing journalism and digital news as socio-technical assemblages, revealing how technologies, professional routines, and institutional arrangements jointly shape journalistic outcomes [15–17]. Recent studies further show that ANT is particularly effective for examining journalistic truth and verification because it enables researchers to trace chains of associations through which truth claims become stabilized, including the mediating roles of documents, platforms, and infrastructures [18,19]. These studies suggest that ANT is well suited to examining how truth emerges through translation processes rather than through linear professional verification alone.

Building on this perspective, the present study applies Actor-Network Theory to investigate how truth is generated, negotiated, and stabilized in Chinese investigative journalism within social media environments. This approach is particularly appropriate in China, where state-regulated media intersect with decentralized platforms, producing hybrid dynamics of control and collaboration [20]. By tracing controversies across platforms, journalistic practices, and institutional interventions, the study seeks to demonstrate how truth emerges as the outcome of negotiations among technological systems, media actors, and governance structures in contemporary digital news ecology.

## Literature review

### China’s online news production

Since the 1980s, Chinese government institutions have promoted media commercialization through reforms to alleviate fiscal pressure. With the expansion of the Internet, television, and online news websites in the late 1990s, information dissemination gradually became integrated into the national informatization strategy. During this period, journalists and audiences developed new spaces for expression enabled by technological innovation, such as online forums, although these spaces remained constrained by ideological boundaries [21,22]. Studies of online dissemination patterns based on historical data from more than one thousand Chinese news portals reveal that hot-spot events, even when first reported by a small news portal, can rapidly spread across the entire network through reposting by major news portals [23]. News production thus reflected a form of controlled liberalization.

The rise of Web 2.0 and social media platforms has changed how journalism works, leading to participatory journalism. “Engagement” has become a buzzword, though research shows that most practices emphasize audience feedback and quantitative metrics rather than substantive participatory contributions [24,25]. In the Chinese context, online journalism has been characterized as hybrid, shaped simultaneously by market competition, professional aspirations, and state-imposed boundaries on permissible discourse [26]. Platforms such as WeChat and Weibo enable decentralized participation, sparking debates on transparency and free speech in China [27]. Research on citizen journalism demonstrates that while real-name registration policies have constrained individual practices, citizens collectively employ social media to influence commercial media agendas and, to some extent, policy discussions, thereby reshaping journalism and the public sphere [28,29].

News production requires substantial investment, and social media platforms have consequently reshaped how news is produced, expressed, and received. Chinese online journalism displays several distinctive features. Studies have shown that digital news practitioners in China tend to adopt non-confrontational strategies in news production, prioritizing data traffic over the public interest [30]. In addition, the discursive construction of online news in China is influenced by multiple factors, including legitimacy concerns and the pursuit of international reputation [31]. Taken together, these findings underscore the complex interplay of technology, politics, media regulation, and individual agency in shaping the landscape of digital news production in China.

### Actor-network theory in journalism studies

In recent years, Bruno Latour’s Actor-Network Theory (ANT) has become a prominent framework in journalism and communication research [32]. The theory has gained wide recognition because it rejects the traditional subject-object dichotomy and offers a distinctive perspective for interactions between human and nonhuman entities. Unlike other network theories, Latour employs “agency” to denote all entities exerting influence in action processes, agency is distributed across both human and nonhuman actants. Agency, mediator, and network constitute the core concepts. ANT posits that all mobilized elements in actions, including human actors and non-human objects, function as mediators. In Latour’s formulation, a network comprises a string of actions, emphasizing processes of work, interaction, flow, and change. Consequently, ANT mandates reinterpreting “actants” anew in each instance, respecting their diversity, and documenting how varied participants translate and construct reality [18].

ANT’s emphasis on continuity and dynamism makes it ideal for examining how technology supports and transforms journalism, thereby challenging and broadening traditional views of news production [17]. Turner [15] pioneered ANT’s application in journalism studies, highlighting its value in identifying and critiquing “hybrid actors that are increasingly becoming a feature of journalism in multimedia environments” (p. 322). Similarly, Couldry [33] views ANT as a tool for analyzing complex power dynamics and actor roles in media networks [19]. Teurlings [34] delved into network connections, role distributions, and actors’ influence on media content to enhance understanding of media power structures. Research on automation in newsrooms reveals that, while journalists position machines as final arbitrators, these technologies play a more transformative role than mere mediation [35]. Nawaarthne and Storni [18] employ ANT to reevaluate news production’s capacity for truth revelation, framing it as a “progressive black box” where truth arises not as a static entity but from “chains of news made of heterogeneous materials and connections” (p. 1647). These applications demonstrate ANT’s value in reassessing journalism’s capacity for truth amid digital disruptions.

This perspective offers a valuable lens for examining the negotiable dynamics of media interactions [16] and equips scholars with a methodological toolkit that prioritizes descriptions of media ecologies over prescriptive models.

### Social media, news and actor-network theory

Since the surge of fake news during the 2016 U.S. presidential election, “post-truth” has become widely discussed. These phenomena have challenged the traditional authority of journalism and reshaped how the public perceives truth. Scholars have pointed out that the spread of fake news reflects deeper tensions in contemporary society, revealing the contested status of journalism and the dynamic processes through which beliefs are formed [14]. It symbolizes the collapse of an older news order and the fragmentation of the modern public sphere. In this context, revisiting the question of news authenticity and understanding the role of social media have become increasingly important.

Social media connect journalists, editors, audiences, and advertisers, forming complex networks in which multiple human and nonhuman actors interact. Platforms such as Weibo and WeChat have introduced decentralized forms of participation, allowing audiences to comment on, share, and evaluate news content. These interactive features influence agenda-setting and shape the construction of authenticity [27]. Studies suggest that such engagement often focuses on visibility and quantitative metrics rather than substantive participation [24,25].

Within this media ecology, social media algorithms and technical architectures operate as nonhuman actors that affect news production, circulation, and reception. Algorithmic recommendation systems tend to prioritize stories that attract high levels of attention and engagement, which may not necessarily align with journalistic accuracy or public interest [36]. As a result, journalists and editors must adapt to platform logic and audience demand, sometimes making compromises in news selection and framing. Meanwhile, audiences’ perceptions of authenticity are shaped by the discursive environment of social media and by the opinions of others, leading to diverse interpretations of what constitutes truth.

In China, these transformations unfold within a hybrid system shaped by market competition, professional norms, and state regulation [26]. Research shows that digital news practitioners often adopt non-confrontational and pragmatic strategies, emphasizing web traffic over public service goals [29]. The discursive construction of news is further influenced by concerns about legitimacy, authority, and international reputation [27]. Under these conditions, social media serve as both participatory spaces and instruments of governance, linking emotional expression with institutional mechanisms of control. To capture the complexity of these interactions, recent scholarship has increasingly drawn on Actor-Network Theory (ANT). In journalism studies, ANT has been used to investigate how digital infrastructures, algorithms, and material devices shape news practices [19,37].

Although actor-network theory is useful when studying investigative journalism, it still has some problems. For instance, while conceptualizing all individuals and entities as “actors” is innovative, it risks oversimplifying the analysis of subjects and may not effectively uncover the institutional power relations, governance arrangements, and structural inequalities that shape news production processes. Additionally, the construction path of actor networks lacks unified standards, making it challenging to develop a general theory across cases. Building on the method of controversy mapping, this study proposes an analytical framework that enables researchers to better trace how actors interact and negotiate with one another. It conceptualizes news authenticity as the relational outcome of translation among multiple actors. This approach offers a useful lens for analyzing the development and legitimization of China’s online news ecology.

Based on the above, this study explored the following questions:

RQ1: Which human and non-human actors are most salient in China’s digital news environment, and how do they interact to shape journalistic practices?

RQ2: In what ways do actor-networks influence the visibility and legitimacy of news, and which nodes function as mediators within these processes?

RQ3: How is “truth” negotiated and produced within actor-networks across China’s social media platforms?

## Methods and materials

### Case selection

The multiple case study method is widely employed to explore complex social phenomena, construct theoretical frameworks, and enhance external validity through cross-case comparisons [38,39]. In this study, case selection followed a theoretically informed comparative logic rather than convenience sampling, ensuring that each case contributes to identifiable analytical dimensions relevant to truth production in digital journalism. The selection of three cases is not arbitrary. Each case anchors a distinct mode of controversy formation in China’s digital news ecology and, taken together, they form a deliberately constructed comparative matrix that reveals how “truth” is produced across heterogeneous domains.

Specifically, the cases were selected based on four criteria: (1) cross-domain variation, ensuring that controversies emerged in distinct social domains, including public safety, celebrity consumption, and educational equity; (2) actor heterogeneity, involving interactions among citizens, journalists, institutions, and platform infrastructures; (3) complete controversy cycles, allowing observation from initial exposure to institutional stabilization; and (4) high platform visibility, ensuring that events triggered large-scale online participation and media intervention.

By analyzing multiple cases, we aim to capture not only the structural dynamics of networks but also the interpretive processes through which truth is constructed. The selected cases span distinct issue domains, public safety, celebrity consumption, and educational equity, while remaining independent of one another. They feature heterogeneous actors, unfold within complete temporal cycles, and have stimulated broad platform-based discussions of social significance, thereby ensuring their representativeness and relevance to the research focus.

Importantly, the three cases also enable comparison across key analytical dimensions, including (1) degrees of platform amplification, and (2) mechanisms through which official institutions ultimately stabilized or redefined truth claims. According to Golden-Biddle and Locke [40] and Pozzebon [41], interpretive case studies are expected to demonstrate authenticity, plausibility, criticality, and reflexivity in order to ensure methodological rigor. We attempted to log all available materials appearing in social networks to reconstruct the trajectories of investigative activities for three events. The three selected cases represent different pathways through which controversies moved from online exposure to institutional resolution, thereby allowing comparison of how actor-networks reorganize around competing truth claims.

We attempted to log all available materials appearing in social networks to reconstruct the trajectories of investigative activities for three events. Case 1 (a child disappearance in Shanghai, 2023). Case 2 (the Jewelry Exposure Case involving Actor Huang, 2025). Case 3 (the college entrance examination migration violation involving Actor Na, 2025). Together, the cases enable comparison between emergencies driven by public anxiety (Case 1), entertainment scandals amplified by consumption and class discourse (Case 2), and institutional controversies concerning policy fairness (Case 3), thus revealing how different social contexts shape truth negotiation processes.

### Analytical procedures

Our data collection strategy was divided into two steps: First, systematically collecting and organizing qualitative materials, including media articles, and official statements, to identify key actors. For each case, we constructed a chronologically ordered narrative depicting the interactions between key events and their actors [32]. According to Latour’s [42] theory and previous research, the method for identifying and determining actors is not a static classification but a dynamic, empirical process that emphasizes “following the actors.” Therefore, we attempted to start from the case phenomena, listing all possible participating entities without presupposing their importance. By observing all discoverable public actions of actors in social media, we collected the actors’ trajectories in time and space and traced associations. Through the processes of following and translation, we constructed the actor network and confirmed actors via cross-source triangulation, ensuring comprehensive capture of heterogeneous networks.

We adopted a structured protocol for data identification, extraction, and analysis across the three cases. The study relied on publicly available data from major national news outlets, and provincial media platforms. The observation window for each case extended from its first online appearance to the point at which official announcements were issued and public discussion significantly declined, as indicated by the disappearance of event-related hashtags.

All retrieved materials were manually archived. Inclusion criteria required that data be: (1) publicly accessible, (2) directly related to the event, and (3) anchored to a verifiable actor or institutional source. Exclusion criteria comprised duplicated posts, missing timestamps, unverifiable rumors, and incomplete screenshots lacking provenance.

Although the four stages of translation provide a theoretical structure, stage identification in this study was guided by observable empirical transitions. In analysis, actors were identified through systematic tracing of publicly observable actions, including posting, commenting, reposting, issuing statements, and producing documentary evidence across platforms, allowing actor participation to be reconstructed through time-stamped interactions rather than predefined classifications. Transitions between stages were often gradual and sometimes overlapping.

### Case description

#### Case 1: the shanghai child beach disappearance.

Case 1 began on October 4, 2023, when four-year-old Huang vanished while playing on a beach in Nanhui New Town, Shanghai. The incident quickly evolved from a local emergency into a nationwide online controversy involving citizens, rescue teams, media organizations, and law enforcement agencies. Surveillance footage, digital maps, and tide simulations functioned as crucial non-human actors in shaping interpretations of the event.

#### Case 2: A teenage actress wearing “expensive earrings” and the legality of her father’s income.

On May 11, 2025, a teenage actress posted photos of her coming-of-age ceremony on social media, triggering online speculation about the legitimacy of her family’s wealth. The controversy rapidly expanded beyond celebrity gossip, becoming a broader debate about class inequality and social justice. Entertainment journalists, regulators, influencers, and platform users participated in constructing competing narratives, while algorithms and digital evidence structured information visibility. This case demonstrates how consumption culture and platform amplification contribute to truth negotiation in entertainment-related controversies.

#### Case 3: Actress suspected of falsifying college entrance examination application materials and breaching training agreement.

On June 8, 2025, online reports alleged that actress Narnaxi had falsified registration materials related to a targeted college entrance program. The controversy centered on issues of educational fairness and policy integrity, prompting widespread scrutiny across media platforms. Official verification processes, media investigations, and platform discussions collectively shaped public interpretations.

Actors were identified by tracing publicly observable actions across platforms, including posting, commenting, reposting, issuing statements, and producing documentary evidence.

## Findings

### Problematization: Making controversies and actors visible

Controversy provides a valuable opportunity to observe society, for it is precisely in such moments that actors become visible and their relationships emerge [43]. Across the three cases, problematization reveals which actors become visible and how initial networks are assembled, thereby addressing RQ1 concerning the identification and interaction of salient human and non-human actors. Before analyzing how each controversy became problematized, Table 1 outlines the discoverable public actions and the initial points of contention associated with each case. These actions serve as early indicators of how individual events were recast as matters of public concern.

**Table 1:** Discoverable public actions and main controversies of three online news cases.

Case	Key Public Actions	Main Controversies
Case 1	Child disappearance reported on Shanghai beach Family circulated missing notices online Surveillance footage analyzed by media and police Volunteer rescue operations organized Police investigation concluded accidental drowning	Timeline inconsistency between parents and surveillance footage Possibility of abduction Tide and terrain safety risks Parental supervision responsibility Transparency of public information
Case 2	Actress posted photos wearing luxury earrings Netizens questioned family wealth sources Father denied corruption allegations Jewelry appraisal report released Disciplinary investigation announced	Authenticity and valuation of earrings Legitimacy of luxury consumption relative to income Boundary between public office and private business Celebrity image and media framing
Case 3	Online allegations of falsified Gaokao registration Media investigated public records Education department verified the case Admission and registration documents examined Public debate on educational fairness intensified	Legality of Gaokao registration Compliance with preferential admission policies Authenticity of admission records and exam scores Educational fairness and privilege

Marres and Moats [44] note that public controversies are spaces where social materiality and technological mediation are intricately intertwined. Problematization marks the moment when a controversy becomes explicit, when dominant explanations can no longer satisfy public demands for interpretation [45]. In other words, problematization is not merely the articulation of a problem; rather, it is a process through which discourse and media entanglements transform specific events into issues that can be socially perceived and acted upon.

In the Shanghai disappearance case, family members used personal media accounts to seek help, turning a private tragedy into a public concern about safety supervision and social rescue mechanisms. The absence of surveillance footage and tidal data at the scene triggered the first wave of public discussion. Subsequently, the father’s ambiguous statements redirected online attention toward questions of parental responsibility, transforming the Weibo hashtag “Shanghai Beach Missing Girl” into a temporary site of controversy.

In the second case, a birthday photo posted on social media sparked discussions about class consumption and the ethics of public officials. Online commentary shifted between verifying the authenticity of the earrings and questioning the legitimacy of political income. In the third case, the Gaokao migration controversy began with an actress’s personal interview, which exposed the gray areas of the education system. News outlets later conducted timeline based investigations into the legality of her university admission.

According to the translation logic, this process represents the core outcome of problematization, defining who has the authority to name the problem and who is required to respond [32]. Dispersed digital traces such as photos, administrative documents, screenshots, and rapidly shifting user emotions gradually converged through platform architectures and circulation practices. Emotional reactions from users created strong adhesion, drawing diverse participants into a shared space of attention. Through this combined work of platforms, users, documents, and news organizations, individual events were reassembled into socially recognizable issues.

During this process, both human and non-human actors, including hashtags, screenshots, and databases, reviewed and challenged the weak points within these authoritative frameworks. In China’s online news environment, the coexistence of official epistemic authority and platform based interpretive communities further intensified this dynamic [20]. Extensive digital data chains enabled users to cross reference official statements, screenshots, and transaction records, creating an alternative evidentiary circuit outside traditional journalistic gatekeeping systems, even though the reliability of such information remained uncertain.

### Interessement: Alliance formation and network intensification

Interessement refers to a set of actions through which network builders seek to impose and stabilize the definitions established during problematization by engaging other participants [42]. At this stage, non-human actors such as evidence, news reports, and policy documents played key mediating roles. They did not merely circulate information but helped connect actors and align competing interpretations. When artifacts such as surveillance clips, screenshots, or official notices were repeatedly shared across platforms, they became focal reference points that reorganized interactions. In doing so, they formed traceable chains of evidence that structured public scrutiny. Social media platforms operated as obligatory passage points, channeling participation through trending lists, topic pages, and comment interfaces [45,46]. Table 2 summarizes the controversy mapping of actor-networks across the three cases, identifying key human and non-human actors, their connections, mediating roles, and translation effects during the development of online controversies.

**Table 2:** Actors across three online news controversies.

Case	Actor	Actor Type	Connected To	Mediation Function	Alliance Type	Translation Effect
Case1	Parents	Human	Netizens, Media	Emotional triggering	Public coalition	Issue visibility amplification
	Surveillance footage	Non-human	Journalists, Police	Evidentiary anchoring	Verification cluster	Redefinition of abduction narrative
	Weibo hashtag	Non-human	Users, Algorithm	Attention aggregation	Platform-driven alliance	Agenda focusing
	Volunteers	Human	Parents, Media	Search execution	Rescue alliance	Moral legitimacy reinforcement
	Police	Human	Media, Public	Preliminary clarification	Authority intervention	Narrative containment
Case2	Netizens	Human	Bloggers, Hashtags	Moral framing	Moral critique cluster	Class discourse amplification
	Appraisal report	Non-human	Journalists, Authorities	Evidence stabilization	Institutional alliance	Luxury narrative reframing
	Bloggers	Human	Users, Screenshots	Interpretive amplification	Media-user coalition	Wealth legitimacy questioning
	Disciplinary authority	Human	Media	Accountability inscription	State regulatory alliance	Closure preparation
Case3	Policy documents	Non-human	Journalists, Bloggers	Interpretive framing	Policy debate cluster	Fairness narrative expansion
	Education Department	Human	Media, Public	Official verification	Governance alliance	Institutional stabilization
	Education Certificate	Non-human	Users, Media	Documentary circulation	Evidence cluster	Legitimacy contestation
	Journalists	Human	Public, Authorities	Timeline reconstruction	Mediation alliance	Issue reframing

In Case 1, the circulation of surveillance footage and rescue updates aligned parents, volunteers, media, and police around the search effort. However, conflicting statements among rescue teams and suspicions directed at the father quickly fragmented this coalition, revealing the instability of alliances grounded in emotional urgency.

In Case 2, comment sections and screenshots transformed a personal image into a moral controversy over wealth legitimacy. Users, bloggers, and journalists formed a media-user coalition that questioned class privilege. The introduction of appraisal reports and regulatory investigation reoriented the network toward institutional accountability.

In Case 3, policy documents and archival records connected whistleblowers, journalists, and education authorities within a debate on fairness. As documentary evidence circulated, alliances were repeatedly reorganized, demonstrating how material artifacts both stabilize and reconfigure interpretive positions.

Across cases, interessement represents the moment of peak relational density. Alliances intensify through shared focal points but remain provisional. New documents, reinterpretations, or institutional interventions can rapidly reorganize network alignments. Rather than producing stability, this stage amplifies interpretive competition before later consolidation.

### Enrollment: Role stabilization and narrative consolidation

Enrollment marks the stage in which actor positions become more clearly defined and network roles are consolidated. Compared to the fluid alliances of interessement, this phase is characterized by stronger evidentiary integration and clearer role differentiation.

In Case 1, rescue teams and volunteers initially gained visibility as executors of the search effort. However, conflicting statements among organizations blurred role boundaries until the police issued a forensic report and official statement. These institutional inscriptions re-centered authority and stabilized a traceable version of events, transforming emotional mobilization into institutional credibility.

In Case 2, bloggers and media accounts acted as narrative mediators, framing the controversy around wealth legitimacy through screenshots and value comparisons. As appraisal reports and disciplinary notices entered the network, interpretive competition narrowed. Institutional documentation reorganized the debate into a formal accountability framework.

In Case 3, policy documents and media investigations structured the controversy into a discussion of educational fairness. Editorial commentary and fact-checking activities consolidated dispersed claims into a coherent evidentiary chain. The subsequent intervention of the education department formalized this consolidation through official statements and rectification notices.

Across cases, enrollment represents the transition from interpretive competition to structured role assignment. Documentary artifacts and institutional actors gained centrality, reducing ambiguity and consolidating the network around more stable narrative configurations.

### Mobilization: Stabilizing representations and producing public truths

Mobilization represents the stage in which actor-networks are consolidated through stabilized representations and delegated authority [45,47]. Certain actors begin to speak on behalf of others, and institutional inscriptions transform contested interpretations into administratively recognized facts. Table 3 summarizes the empirical findings across the three cases and outlines the actor configurations and relational mechanisms at each stage of the ANT translation process.

**Table 3:** The four-stage translation process in Chinese online news controversies.

Stage	Human Actors	Non-Human Actors	Primary Relational Mechanism	Network Effect
Problematization	Parents, Netizens	Hashtags, Screenshots	Emotional amplification	Issue visibility expansion
Interessement	Journalists, Bloggers	Evidence fragments, Platform algorithms	Alliance formation & interpretive competition	Peak network density
Enrollment	Media institutions, Experts	Timeline reconstruction, Appraisal reports	Role assignment & narrative framing	Structural consolidation
Mobilization	Government authorities	Official documents, Rectification notices	Institutional inscription	Centralized stabilization

Across the three cases, stabilization occurred through bureaucratic documentation and visibility control. Police reports, disciplinary notices, and official statements translated dispersed narratives into institutional discourse, while platform algorithms privileged authoritative accounts and reduced competing interpretations. Through this combined mechanism of inscription and visibility management, “truth” was produced as a coordinated institutional representation.

In Case 1, the police forensic report re-centered authority and closed interpretive ambiguity. In Case 2, disciplinary investigations formalized accountability and reframed moral criticism into regulatory outcomes. In Case 3, official verification and rectification notices transformed debates on fairness into matters of administrative correction. In each instance, public speculation was absorbed into institutional procedures.

Mobilization thus marks the transition from contestation to governable knowledge. Authority becomes centralized, interpretive plurality declines, and the network stabilizes around institutional narratives.

## Discussion

This study examines how “truth” is produced and stabilized in China’s digital news ecology through the analysis of three online controversies. Drawing on ANT and controversy mapping, the research traces how dispersed signals circulating on digital platforms are gradually translated into institutionally recognized narratives. The findings demonstrate that truth production in contemporary Chinese online journalism does not follow a linear path from event to verified fact. Instead, it emerges through a dynamic process of negotiation among heterogeneous actors, including users, journalists, platform infrastructures, documentary artifacts, and administrative authorities.

Across the three cases, the empirical analysis reveals a translation process that broadly follows the stages described in Actor-Network Theory: problematization, interessement, enrollment, and mobilization. Each stage reflects a different moment in the development of online controversies. Private experiences first emerge as public issues. Actors then form temporary alliances around competing interpretations. Over time, chains of evidence help stabilize certain narratives. Finally, institutions intervene and reframe the controversy through authoritative accounts. Based on these observations, this study proposes a four stage analytical framework inspired by ANT. The framework helps explain how truth is assembled in platform based news environments.

The findings resonate with a growing body of research suggesting that news production in digital environments is shaped by interactions among media institutions, technological infrastructures, and networked publics. Previous studies show that online platforms reshape journalistic practice by structuring information visibility through algorithmic ranking, enabling user participation, and accelerating the circulation of content [48,49]. These mechanisms influence which issues gain attention and enter the news agenda. The cases examined here illustrate how user generated signals, such as screenshots, documents, or emotional expressions, can quickly gain visibility through platform architectures and trigger broader public attention.

The findings also extend existing scholarship. They highlight the central role of documentary artifacts and administrative records in stabilizing contested narratives. Much of the literature on digital journalism focuses on platforms and audience participation [50,51]. However, the present analysis shows that institutional actors remain crucial in determining how controversies eventually conclude. These observations are consistent with research on China’s digital governance model. These arrangements combine algorithmic management with regulatory oversight. The trajectories observed in the three controversies reflect this pattern. Online publics initially amplify issues through emotional resonance and rapid circulation across networks. Government institutions later intervene through bureaucratic records and narrative reframing. These interventions help stabilize interpretations. In this sense, truth production in Chinese digital journalism emerges from the interaction between participatory communication practices and institutional authority.

Beyond confirming patterns identified in previous research, one notable finding concerns the role of non human actors in shaping the trajectory of controversies. In all three cases, documentary artifacts played a decisive role in reorganizing public interpretations. These materials included administrative records, forensic reports, purchase receipts, and surveillance footage. Once such materials entered online circulation, they often became focal points for discussion. Competing narratives were then constructed around them.

Another observation concerns the affective dynamics of online participation. Emotional expressions such as anger, sympathy, and moral outrage frequently acted as catalysts. They mobilized communities and expanded the visibility of controversies. They encouraged users to search for evidence, reinterpret events, and form temporary alliances with others.

Finally, the analysis shows that institutional closure did not necessarily eliminate contestation. Even after official explanations were issued, alternative interpretations often continued to circulate across digital platforms. These interpretations appeared in fragmented forms and persisted in online discussions. This pattern suggests that stabilization in online controversies is better understood as a temporary reorganization of interpretive authority, rather than the complete resolution of disagreement.

### Reflections

The study extends the application of ANT within journalism studies by providing an empirically grounded account of how truth is assembled across interconnected technological and institutional systems [32,52]. More broadly, the findings suggest that contemporary journalism should be understood as a technocultural practice in which credibility and news value are co-produced through interactions among human actors and material infrastructures. Rather than being solely generated within professional newsrooms, factual authority emerges through distributed processes of verification, circulation, and institutional recognition. This perspective helps explain why controversies in digital media environments often evolve through complex cycles of amplification, reinterpretation, and stabilization.

## Conclusions

Across three controversies concerning public safety, elite consumption, and educational equity, this study shows that “truth” in Chinese online journalism is not simply discovered. Instead, it is translated through the continuous interaction of actors. The analysis identifies which actors become most visible in digital controversies and how their interactions shape journalistic practices and public interpretations.

Social media platforms function as key passage points in this process. They amplify emotion and public scrutiny through networked participation. At the same time, algorithmic visibility structures which information gains attention. Bureaucratic documents, including police notices and rectification statements, also play an important role. These documents transform dispersed information into temporary institutional conclusions.

Tracing the stages of problematization, interessement, enrollment, and mobilization reveals a dual process. Citizens generate alternative evidentiary circuits through screenshots, timelines, and transaction records. At the same time, administrative texts reintegrate these materials into frameworks of governance. Through these interactions, actor networks shape both the visibility and the perceived legitimacy of news events. They also influence how truth is negotiated and provisionally stabilized across platform environments.

The findings frame Chinese online news as a technocultural assemblage. In this assemblage, credibility emerges through ongoing negotiation among journalists, users, platforms, and authorities. The temporality, affect, and ethics of journalism are reshaped through socio technical mediation. Methodologically, the combination of Actor Network Theory and controversy mapping helps reveal the evidentiary chains and role translations that construct and stabilize facts. At the same time, it exposes constraints created by platform opacity, selective deletion, and unequal capacities for documentation. In this sense, the study highlights the ongoing negotiation between platform participation and institutional governance as a defining feature of contemporary digital news production. By tracing controversies across platforms and institutions, the study also demonstrates how ANT can be operationalized empirically in journalism research rather than remaining purely conceptual.

## Supporting information

S1 The coded event timeline used in the study, with the case identifier, event identifier, date, time, event description, information source, source URL, and a brief summary of each documented event. (XLSX)
